# Classification tree analysis to evaluate the most useful magnetic resonance image type in the differentiation between early and progressed hepatocellular carcinoma

**DOI:** 10.1002/cam4.5589

**Published:** 2023-01-22

**Authors:** Fumihito Ichinohe, Daisuke Komatsu, Akira Yamada, Takanori Aonuma, Ayumi Sakai, Marika Shimizu, Masahiro Kurozumi, Akira Shimizu, Yuji Soejima, Takeshi Uehara, Yasunari Fujinaga

**Affiliations:** ^1^ Department of Radiology Shinshu University School of Medicine Matsumoto Nagano Japan; ^2^ Division of Gastroenterological, Hepato‐Biliary‐Pancreatic, Transplantation and Pediatric Surgery, Department of Surgery Shinshu University School of Medicine Matsumoto Nagano Japan; ^3^ Department of Laboratory Medicine Shinshu University School of Medicine Matsumoto Nagano Japan

**Keywords:** classification tree, early hepatocellular carcinoma, magnetic resonance imaging, progressed hepatocellular carcinoma

## Abstract

**Aim:**

Using classification tree analysis, we evaluated the most useful magnetic resonance (MR) image type in the differentiation between early and progressed hepatocellular carcinoma (eHCC and pHCC).

**Methods:**

We included pathologically proven 214 HCCs (28 eHCCs and 186 pHCCs) in 144 patients. The signal intensity of HCCs was assessed on in‐phase (T1in) and opposed‐phase T1‐weighted images (T1op), ultrafast T2‐weighted images (ufT2WI), fat‐saturated T2‐weighted images (fsT2WI), diffusion‐weighted images (DWI), contrast enhanced T1‐weighted images in the arterial phase (AP), portal venous phase (PVP), and the hepatobiliary phase. Fat content and washout were also evaluated. Fisher's exact test was performed to evaluate usefulness for the differentiation. Then, we chose MR images using binary logistic regression analysis and performed classification and regression tree analysis with them. Diagnostic performances of the classification tree were evaluated using a stratified 10‐fold cross‐validation method.

**Results:**

T1in, ufT2WI, fsT2WI, DWI, AP, PVP, fat content, and washout were all useful for the differentiation (*p* < 0.05), and AP and T1in were finally chosen for creating classification trees (*p* < 0.05). AP appeared in the first node in the tree. The area under the curve, sensitivity and specificity for eHCC, and balanced accuracy of the classification tree were 0.83 (95% CI 0.74–0.91), 0.64 (18/28, 95% CI 0.46–0.82), 0.94 (174/186, 95% CI 0.90–0.97), and 0.79 (95% CI 0.70–0.87), respectively.

**Conclusions:**

AP is the most useful MR image type and T1in the second in the differentiation between eHCC and pHCC.

## Graphical abstract

Using classification tree analysis, we aimed to evaluate the most useful of magnetic resonance (MR) image type in differentiating between early and progressed hepatocellular carcinoma. The arterial phase image is the most useful MR image type, and T1‐weighted images in phase the second.

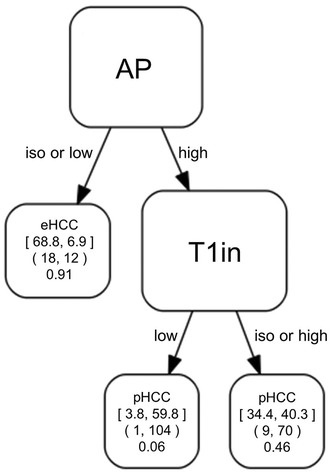

## 1 INTRODUCTION

Hepatocellular carcinoma (HCC) is the fourth most common cause of cancer‐related death worldwide,^1^ and much has been revealed about its pathology in recent decades. HCC develops in a multistep process of hepatocarcinogenesis from dysplastic nodule to early HCC (eHCC) and finally to progressed HCC (pHCC). Consensus regarding the pathological diagnostic criteria of eHCC was reached at the International Consensus Group for Hepatocellular Neoplasia in 2009. The eHCC is vaguely nodular and characterized by various combinations of the following major histologic features: increased cell density and nuclear/cytoplasm ratio, irregular thin‐trabecular pattern, intratumoral portal tracts, pseudoglandular pattern, diffuse fatty change, and varying numbers of unpaired arteries.^2^

The differentiation between eHCC and pHCC is clinically important for two reasons. First, it becomes possible to predict prognosis prior to treatment. The prognosis of eHCC patients undergoing radiofrequency ablation and surgery is better than that of pHCC patients.^3^, ^4^ Furthermore, there remains room for discussion on treatment policy. One study has indicated that the survival benefit of surgery in eHCC is marginal, and that patients with eHCC should be closely observed because the livers of patients with HCC are often damaged.^4^ Percutaneous biopsies can be performed for differentiating eHCC and pHCC prior to treatment. However, they are sometimes technically difficult, and there is a risk of complications such as bleeding or dissemination.^5^, ^6^ Therefore, diagnostic imaging is clinically important in the differentiation between eHCC and pHCC.

Liver Imaging Reporting and Data System (LI‐RADS) is a well‐known system for diagnosing HCC.^7^ However, it does not differentiate between eHCC and pHCC. HCCs with different histological grades sometimes belong to the same class.^8^ Therefore, we need to establish a new approach to differentiate between eHCC and pHCC.

For detection of eHCC, dynamic contrast‐enhanced magnetic resonance imaging (DCE‐MRI) using Gadolinium ethoxybenzydiethylenetriamine pentaacetic acid (gadoxetic acid, Gd‐EOB‐DTPA; Primovist, Bayer Schering Pharma) is more useful than intravenous contrast‐enhanced CT and CT during hepatic arteriography/arterial portography.^8^, ^9^ Differences in signal intensity (SI) between eHCC and pHCC on T1‐weighted images (T1WI), T2‐weighted images (T2WI), diffusion‐weighted images (DWI), arterial phase (AP) images, and the presence of fat evaluated by SI on T1WI (in‐phase and opposed‐phase) have been reported.^8^, ^9^ However, it is unclear how to diagnose eHCC with these MR images. It is necessary to reveal which MR images should be put more importance on for efficient diagnosis, because there are many different types of MR images.

Classification tree is a kind of prediction model in machine learning that take the form of yes or no questions. One of its advantages is to produce human‐readable rules regarding how to classify a given example.^10^ Classification tree analysis (CLTA) has been shown to be effective for the differentiation between dysplastic nodules and HCC,^11^ and it may be effective to solve the present problem. The purpose of this study is to evaluate the most useful MR image type in the differentiation between eHCC and pHCC through CLTA.

## 2 METHODS

### 2.1 Study population

**FIGURE 1:**
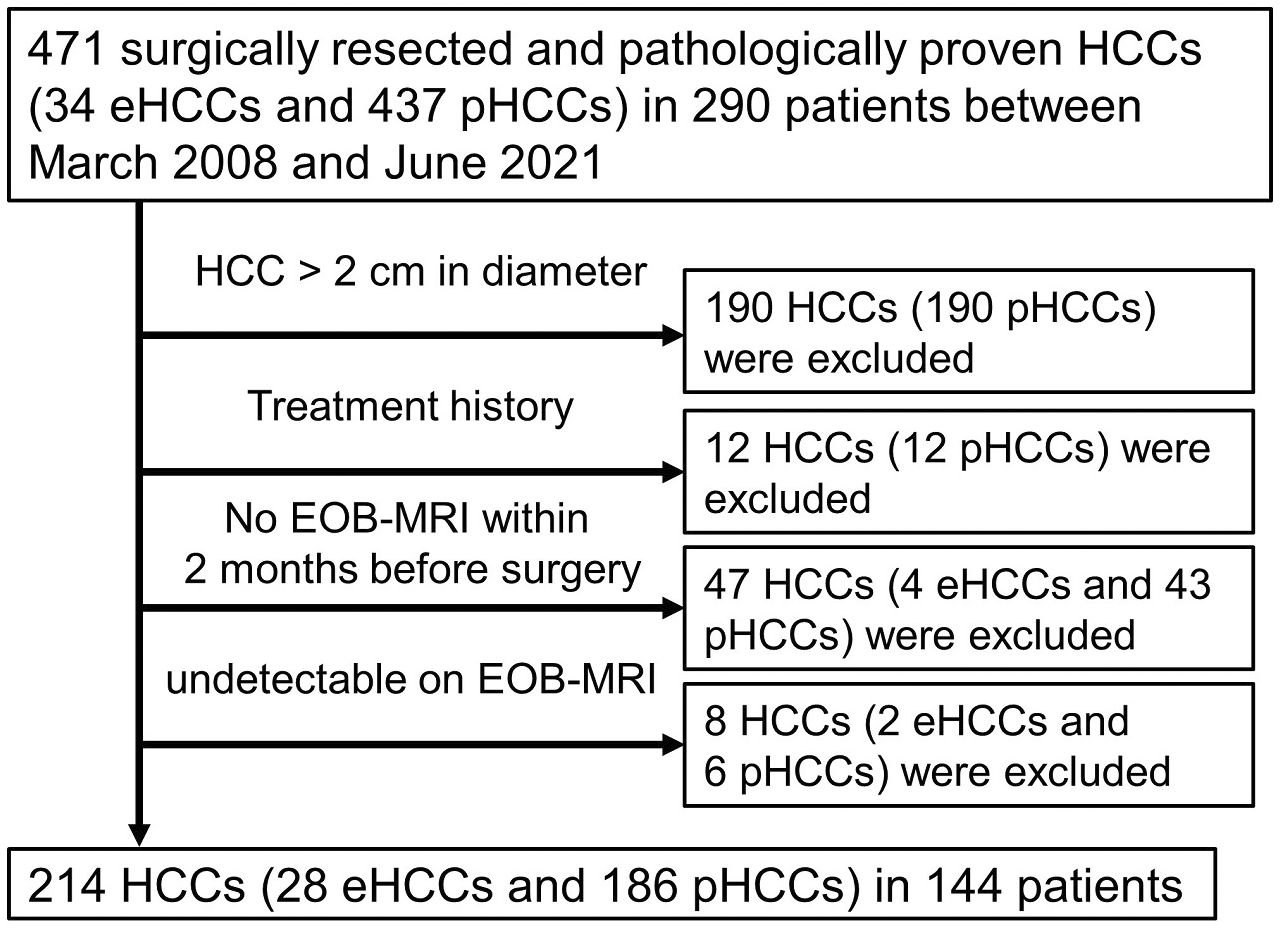
Flow diagram of the study's inclusion and exclusion criteria. eHCC, early hepatocellular carcinoma; EOB‐MRI, Gd‐EOBDTPA‐enhanced magnetic resonance image; pHCC, progressed hepatocellular carcinoma.

This retrospective study was approved by our Institutional Review Board, and the requirement for informed consent was waived. We reviewed our institution's pathology records between March 2008 and June 2021, and 471 pathologically proven HCCs through liver resection (34 eHCCs and 437 pHCCs) in 290 patients were enrolled in this study. Both eHCC and pHCC were diagnosed according to the General Rules for the Clinical and Pathological Study of Primary Liver Cancer (5th and 6th editions) and the criteria of International Consensus Group for Hepatocellular Neoplasia. Among these lesions, 190 HCCs (190 pHCCs) that were over 2 cm in diameter were excluded, because eHCC is usually 2 cm in diameter or less.^12^, ^13^ In addition, 12 HCCs (12 pHCCs) with treatment histories, 47 HCCs (4 eHCCs and 43 pHCCs) without Gd‐EOB‐DTPA‐enhanced magnetic resonance imaging (EOB‐MRI) within 2 months before the surgery, and 8 HCCs (2 eHCCs and 6 pHCCs) that were undetectable on EOB‐MRI were excluded. Finally, 214 HCCs (28 eHCCs and 186 pHCCs) in 144 patients were analyzed in this study (Figure 1). Among the pHCCs, there were 43 well‐differentiated, 104 moderately differentiated, and 39 poorly differentiated HCCs. The patients' backgrounds are shown in Table 1.

**TABLE 1:** Patients' backgrounds

	eHCC (28 HCCs in 23 patients)	pHCC (186 HCCs in 131 patients)	*p* value
Gender (M/F)	18/5	89/42	0.462^a^
Age (years)	41–82 (72)	41–89 (71)	0.724^b^
Etiology of liver disease (HBV/HCV/HBV + HCV/others)	3/11/0/9	28/60/2/41	(HBV/non‐HBV) 0.411^a^ (HCV/non‐HCV) 1.000^a^
Child‐Pugh (A/B)	22/1	123/8	1.000^a^
Duration between EOB‐MRI and surgery (days)	3–54 (26)	2–60 (19)	0.054^b^
Tumor size (mm)	2–20 (13)	2–20 (15)	0.081^b^

*Note*: In total, 10 patients had both eHCC and pHCC. The medians of age, duration between EOB‐MRI and surgery, and tumor size are shown in parentheses.

Abbreviations: eHCC, early hepatocellular carcinoma; EOB‐MRI, Gd‐EOB‐DTPA‐enhanced MRI; F, female; HBV, hepatitis B virus; HCV, hepatitis C virus; M, male; pHCC, progressed hepatocellular carcinoma.

^a^ Fisher's exact test was performed.

^b^ Wilcoxon rank sum test was performed.

### 2.2 MR image acquisition

MR imaging was performed on 140 HCCs using a 3‐Tesla (T) system (MAGNETOM Trio, Siemens Healthcare), on 25 HCCs using a 3‐T system (MAGNETOM Prisma, Siemens Healthcare), on 19 HCCs using a 3‐T system (MAGNETOM Vida, Siemens Healthcare), on 19 HCCs using a 1.5‐T system (MAGNETOM Avanto, Siemens Healthcare, Erlangen, Germany), on 10 HCCs using a 1.5‐T system (MAGNETOM Avanto fit, Siemens Healthcare), on 1 HCC using a 1.5‐T system (Optima MR450w, GE Healthcare, Milwaukee, Wisconsin, the United States of America). First, ultrafast half‐Fourier acquisition single‐shot turbo spin‐echo or single‐shot fast spin echo T2‐weighted images (ufT2WI) were obtained, followed by fat‐suppressed fast/turbo spin‐echo T2‐weighted images (fsT2WI), diffusion‐weighted images (DWI), and T1‐weighted images (T1WI) [in‐phase (T1in) and opposed‐phase (T1op)]. FsT2WI and DWI were obtained after DCE‐MRI in some cases. The MRI scanning parameters are shown in Data S1.

DCE‐MRI was obtained using the standard dose of Gd‐EOB‐DTPA (0.025 mmol/kg body weight) injected at a rate of 2 ml/s followed by 50 ml of 0.9% saline at the same rate. The pre‐contrast image (Pre) was obtained before contrast agent injection. The DCE‐MRI was obtained using the fluoroscopic triggering method (FLTM) in 60 HCCs and the fixed time method (FTM) with multiple phase images in 154 HCCs. From the multiple arterial phase FTM images, two radiologists (25 and 5 years of experience in abdominal imaging, respectively) selected late arterial phase images (defined by hepatic artery enhancement, portal vein enhancement with laminar flow, and faint hepatic parenchymal enhancement) because late arterial phase images are strongly preferred for evaluation of HCC.^7^ They also selected portal venous phase images (PVP) defined by full enhancement in portal veins, enhancement by antegrade flow in hepatic veins, and peak enhancement in liver parenchyma.^7^ Hepatobiliary phase (HBP) images were obtained 15–27 min after injection of Gd‐EOB‐DTPA.

### 2.3 Image analysis

Above‐mentioned two radiologists who were blinded to the patients' pathological findings qualitatively determined whether lesions were high‐, iso‐, or low‐intensity compared with the adjacent liver parenchyma. The presence of fat was considered positive when the SI on T1op was lower than that on T1in. The presence of washout was considered positive in any enhancing observation when temporal reduction in enhancement in whole or part relative to composite liver tissue from AP to PVP.^7^ Disagreement was resolved by discussion and consensus. To evaluate the inter‐reader agreement between the two radiologists, Cohen's kappa coefficient was calculated. A kappa value of ≤0.20 indicated poor agreement; 0.21–0.40, fair agreement; 0.41–0.60, moderate agreement; 0.61–0.80, good agreement; and 0.81–1.00, excellent agreement.

### 2.4 Classification tree analysis and statistical analysis

The CLTA and statistical analysis consisted of three steps. The first step was to determine the MR image types for creating classification trees. The useful MR image types for differentiating between eHCCs and pHCCs were analyzed using Fisher's exact test after making a 2 × 2 table. Diagnostic performance metrics such as sensitivity and specificity for eHCC, and balanced accuracy were also calculated. Then, binary logistic regression analysis was performed using variables with a *p* value <0.05 at Fisher's exact test in order to choose MR image types for creating classification trees.

The second step was to create the classification tree with MR image types chosen in the first step. Classification and regression tree analysis using the stratified 10‐fold cross‐validation method was performed. We performed 100 trials and chose the trial with the 50th highest AUC in order to avoid the effects of data splitting in the cross‐validation method.

There were not enough cases to construct a validation set to optimize hyperparameters. We therefore set the minimum number of cases in a leaf to 10% of the total number of cases so that we obtain reproducible trees between folds. If we make more complex trees, trees obtained were different between folds in the cross‐validation method. The ratio of eHCC to pHCC was 0.15, and when one class is rare in a classification problem, trees often do not work well at diagnosing that class. Therefore, we set “class_weight” (a hyperparameter in Scikit‐learn) to “balanced” to automatically adjust the class weights to be inversely proportional to the class proportions in the training dataset.

The third step was to assess diagnostic performance metrics of the tree: area under the curve (AUC), sensitivity and specificity for eHCC, balanced accuracy. We calculated 95% confidence interval (95% CI) for them using 2000 iterations of the stratified bootstrap method. The diagnostic probability of a case assigned by a classification tree is the class proportion of the leaf node to which the case is assigned. We did not average the AUC values of each fold in the cross‐validation method because each test set contained only two or three eHCCs. Instead, we calculated the overall AUC (AUC merge): to sort the individual scores from all folds together into a single ROC curve and then compute the AUC.^14^

To evaluate the usefulness of the above‐mentioned tree, we performed two comparisons. First, we compared the AUC of the above‐mentioned tree to that of the tree created with the MR image in the first node by itself. Second, we calculated the AUC values for all combinations of useful MR image types chosen by Fisher's exact test in the first step. We compared the maximum AUC values among them to that of the above‐mentioned tree. The AUC comparison was performed using Delong test.^15^

The binary logistic regression was performed using the vcd package,^16^ and the AUC comparison was performed using the pROC package^17^ implemented in R version 4.0.2 (R Foundation for Statistical Computing). The other analyses were performed using the Scikit‐learn version 0.22.2 package or statsmodels version 0.12.1 package implemented in Python version 3.7.3 (Python Software Foundation). A *p* value <0.05 was considered to indicate statistical significance.

## 3 RESULTS

### 3.1 The MR images for creating the classification tree

The inter‐reader agreement between the two radiologists was good or excellent: Cohen's kappa coefficients were 0.84 for T1in, 0.85 for T1op, 0.77 for fat, 0.95 for ufT2WI, 0.89 for fsT2WI, 0.91 for DWI, 0.84 for AP, 0.82 for PVP, 0.77 for washout, and 0.91 for HBP.

The SI on T1in, ufT2WI, fsT2WI, DWI, AP, PVP and the presence of washout and fat were all significantly useful for the differentiation (*p* < 0.05, Table 2). Diagnostic performance of each MR image type is shown in Table 3. AP and T1in were finally chosen through binary logistic regression (*p* < 0.05, Table 2).

**TABLE 2:** The results of Fisher's exact test and binary logistic regression analysis

MR image type	SI	eHCC (*n* = 28)	pHCC (*n* = 186)	Fisher's exact test *p* value	Binary logistic regression analysis	
					Odds ratio (95% CI)	*p* value
T1in	low iso, high	3 25	108 78	*<0.001*	4.85 (1.18–26.15)	*0.040*
T1op	low iso, high	15 13	131 55	0.084	—	—
Fat	absent present	13 15	139 47	*0.003*	2.96 (0.89–10.62)	0.083
ufT2WI	low, iso high	16 12	43 143	*<0.001*	0.78 (0.19–3.51)	0.732
fsT2WI	low, iso high	16 12	17 169	*<0.001*	1.75 (0.28–11.79)	0.552
DWI	low, iso high	15 13	23 163	*<0.001*	0.70 (0.20–2.74)	0.588
AP	low, iso high	18 10	12 174	*<0.001*	0.04 (0.01–0.18)	*<0.001*
PVP	low iso, high	15 13	136 50	*0.045*	1.66 (0.20–10.58)	0.607
Washout	absent present	13 15	44 142	*0.020*	1.15 (0.15–6.97)	0.887
HBP	low iso, high	24 4	174 12	0.237	—	—

*Note*: Odds ratio of being eHCC in the presence of signal intensity in the lower rows had been shown.

Abbreviations: 95%CI, 95% confidence interval; AP, arterial phase image; DWI, diffusion‐weighted image; eHCC, early hepatocellular carcinoma; fsT2WI, fat‐suppressed fast/turbo spin‐echo T2‐weighted image; HBP, hepatobiliary phase image; pHCC, progressed hepatocellular carcinoma; PVP, portal venous phase image; SI, signal intensity; T1in, in‐phase T1‐weighted image; T1op, opposed‐phase T1‐weighted image; ufT2WI, ultrafast half‐Fourier acquisition single‐shot turbo spin‐echo or single‐shot fast spin echo T2‐weighted image.

**TABLE 3:** Diagnostic performance for eHCC of each MR image type

	Diagnosis	Sensitivity (95% CI)	Specificity (95% CI)	Balanced accuracy (95% CI)
T1in	low: pHCC iso, high: eHCC	0.89 (0.79–1.00)	0.58 (0.51–0.65)	0.74 (0.66–0.80)
T1op	low: pHCC iso, high: eHCC	0.46 (0.29–0.64)	0.70 (0.64–0.77)	0.58 (0.49–0.68)
Fat	absent: pHCC present: eHCC	0.54 (0.36–0.71)	0.75 (0.68–0.81)	0.64 (0.54–0.74)
ufT2WI	low, iso: eHCC high: pHCC	0.57 (0.39–0.75)	0.77 (0.70–0.83)	0.67 (0.57–0.76)
fsT2WI	low, iso: eHCC high: pHCC	0.57 (0.39–0.75)	0.91 (0.87–0.95)	0.74 (0.65–0.83)
DWI	low, iso: eHCC high: pHCC	0.54 (0.36–0.71)	0.88 (0.82–0.92)	0.71 (0.61–0.80)
AP	low, iso: eHCC high: pHCC	0.64 (0.46–0.82)	0.94 (0.90–0.97)	0.79 (0.70–0.87)
PVP	low: pHCC iso, high: eHCC	0.46 (0.29–0.64)	0.73 (0.67–0.80)	0.60 (0.50–0.70)
Washout	absent: eHCC present: pHCC	0.46 (0.29–0.64)	0.76 (0.70–0.82)	0.61 (0.51–0.71)
HBP	low: pHCC iso, high: eHCC	0.14 (0.04–0.29)	0.94 (0.90–0.97)	0.54 (0.48–0.61)

Abbreviations: AP, arterial phase image; DWI, diffusion‐weighted image; eHCC, early hepatocellular carcinoma; fsT2WI, fat‐suppressed fast/turbo spin‐echo T2‐weighted image; HBP, hepatobiliary phase image; pHCC, progressed hepatocellular carcinoma; PVP, portal venous phase image; T1in, in‐phase T1‐weighted image; T1op, opposed‐phase T1‐weighted image; ufT2WI, ultrafast half‐Fourier acquisition single‐shot turbo spin‐echo or single‐shot fast spin echo T2‐weighted image.

### 3.2 The classification tree obtained and its diagnostic performance metrics

**FIGURE 2:**
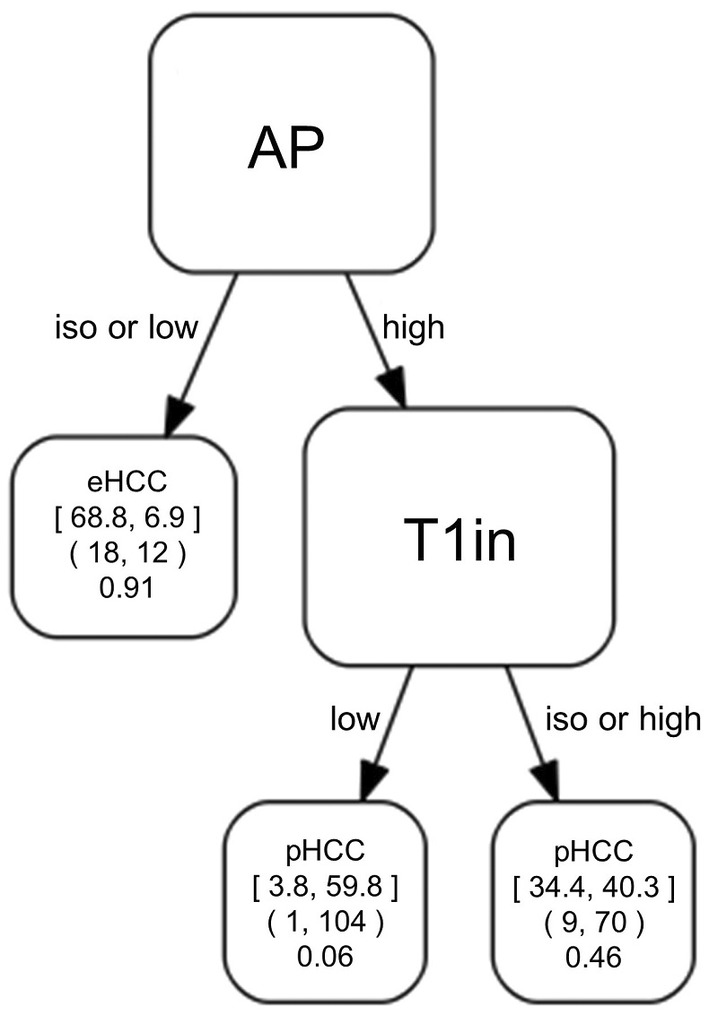
The classification tree produced by the combination of AP and T1in The left and right numbers in the square brackets denote the adjusted counts of eHCCs and pHCCs in that node, respectively. The left and right numbers in the parenthesis denote the actual counts of eHCCs and pHCCs in that node, respectively. The average probabilities of being eHCC are shown below the parenthesis. AP, arterial phase image; T1in, in‐phase T1‐weighted image; eHCC, early hepatocellular carcinoma; pHCC, progressed hepatocellular carcinoma

The median, average, and standard deviation of AUC were 0.83, 0.82, and 0.01 in the 100 trials. The combination of AP and T1in produced the tree as shown in Figure 2, and AP appeared in the first node. In the tree, HCCs that show low‐ or iso‐ intensity on AP are diagnosed as eHCC. HCCs that show high intensity on AP are diagnosed as pHCC, and in those cases low intensity on T1in indicates higher probability of pHCC. The AUC, sensitivity and specificity for eHCC, and balanced accuracy were 0.83 (95% CI 0.74–0.91), 0.64 (18/28, 95% CI 0.46–0.82), 0.94 (174/186, 95% CI 0.90–0.97), and 0.79 (95% CI 0.70–0.87) respectively.

The AUC value of the combination of AP and T1in was significantly higher than that of AP (0.70, 95% CI 0.56–0.84, *p* = 0.003). The maximum AUC among all combinations of useful MR image types chosen by Fisher's exact test was obtained with a combination of AP, T1in, and PVP (0.85, 95% CI 0.77–0.93). However, there was no significant difference between this and the AUC value of the combination of AP and T1in (*p* = 0.173). Receiver operating characteristic curves are shown in Figure 4.

## 4 DISCUSSION

The T1in, ufT2WI, fsT2WI, DWI, AP, PVP, washout, and fat were all significantly useful for the differentiation between eHCC and pHCC. The SI of HCC on MR images changes during the multistep process of hepatocarcinogenesis: high intensity on T1WI is attributed to steatosis, clear cell formation, and the presence of copper^18^; high intensity on T2WI is attributed to intratumoral dilated sinusoids^19^; high intensity on DWI is attributed to cell density and architectural complexity^20^; and high intensity on AP is attributed to arterial blood supply.^21^, ^22^ There were statistically significant differences in PVP and washout contrary to previous studies.^8^, ^9^ However, PVP was obtained 60 seconds after the administration of contrast materials in these studies, and there might be not optimal PVP as defined in LI‐RADS‐v2018.^7^ Furthermore, differences in PVP and washout are reasonable, because intratumoral portal tracts is one of important histologic features of eHCC compared to pHCC.^2^ T1in was significantly useful for the differentiation, but T1op was not. This might be because the SI of eHCCs on T1op was decreased by their fat. HBP was not significantly useful, although it has been reported that the decrease in uptake of Gd‐EOB‐DTPA occurs during hepatocarcinogenesis.^23^ This might be because we evaluated the SI qualitatively, while the previous study evaluated quantitatively. Both eHCCs and pHCCs show low intensity compared to the surrounding liver.

CLTA revealed that AP is the most useful MR image in the differentiation between eHCC and pHCC. This is reasonable, because more portal tracts and fewer unpaired arteries are important pathological features of eHCC when compared to pHCC.^2^, ^12^, ^13^

**FIGURE 3:**
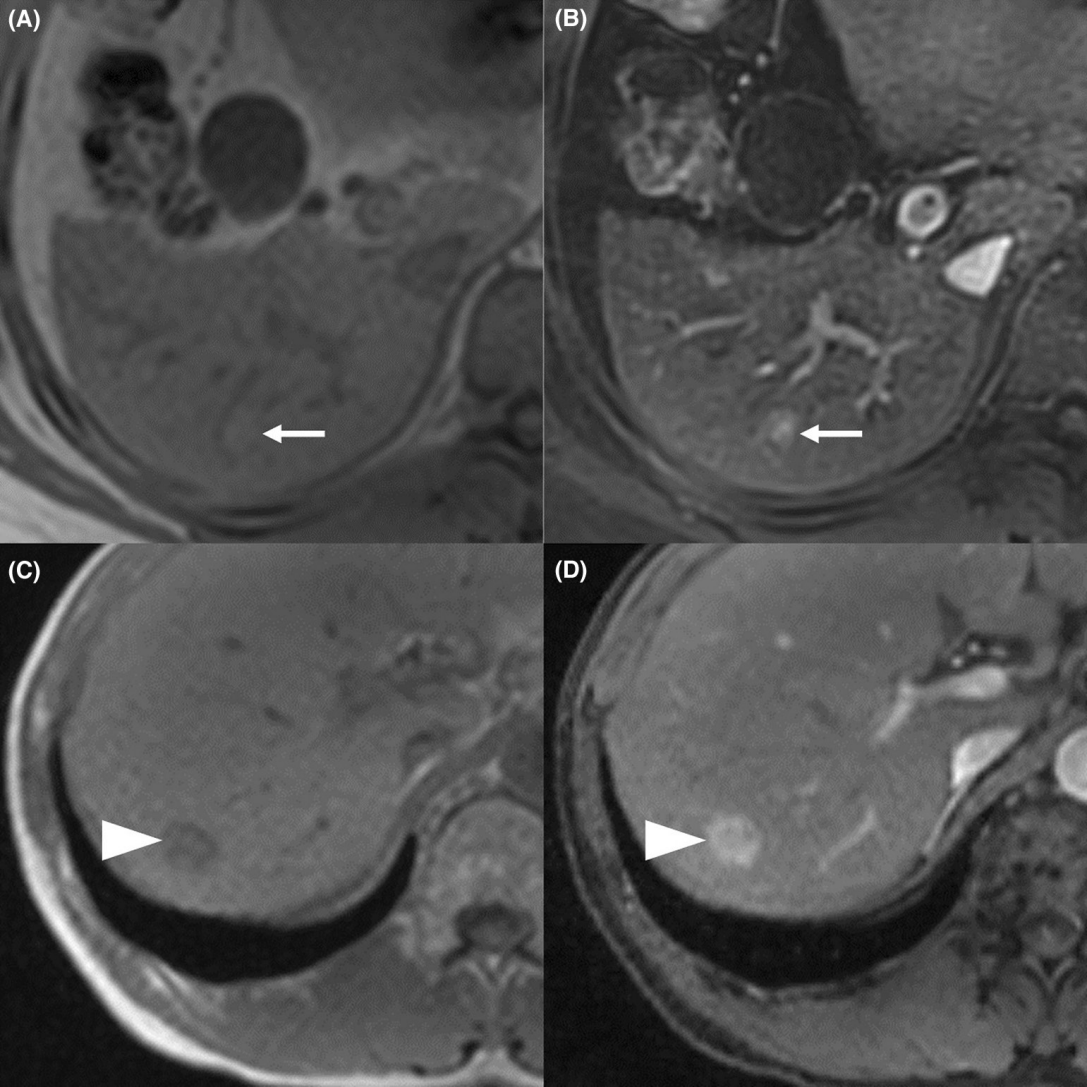
An advantage of the addition of T1in to AP In‐phase T1‐weighted image (A) and arterial phase image (B) of eHCC in a 46‐year‐old male patient, and in‐phase T1‐weighted image (C) and arterial phase image (D) of pHCC in a 56‐year‐old male patient are shown. Both HCCs show high intensity on arterial phase images. However, the eHCC (arrows) shows iso‐ to slightly high intensity, and the pHCC (arrowheads) shows low intensity on in‐phase T1‐weighted image. AP, arterial phase image; T1in, in‐phase T1‐weighted image

The addition of T1in to AP significantly improved the AUC compared with using AP by itself. The classification tree of AP diagnosed high‐intensity HCC as pHCC and iso‐ or low‐intensity HCC as eHCC. Only a few pHCCs showed iso‐ or low intensity (6% in our study), but some eHCCs showed high intensity (36% in our study). It is important to diagnose eHCCs that show high intensity on AP, but we could not find a successful combination of MR images in our study. Instead, the tree of AP and T1in diagnosed two types of HCCs as pHCC, but with different probabilities (Figure 2), and improved the AUC value: (i) HCC with high intensity on AP and low intensity on T1in, and (ii) HCC with high intensity on AP and iso‐ or high intensity on T1in (Figure 3). In other words, HCCs that show high intensity on AP and low intensity on T1in are almost certainly pHCC. There might be two reasons that these findings are crucial. One is that eHCCs seldom show low intensity on T1in (11% in our study, 0%–19% in previous studies^8^, ^9^, ^24^). The other is that the presence of low intensity on T1in along with high intensity on AP implies marked arterial enhancement.

**FIGURE 4:**
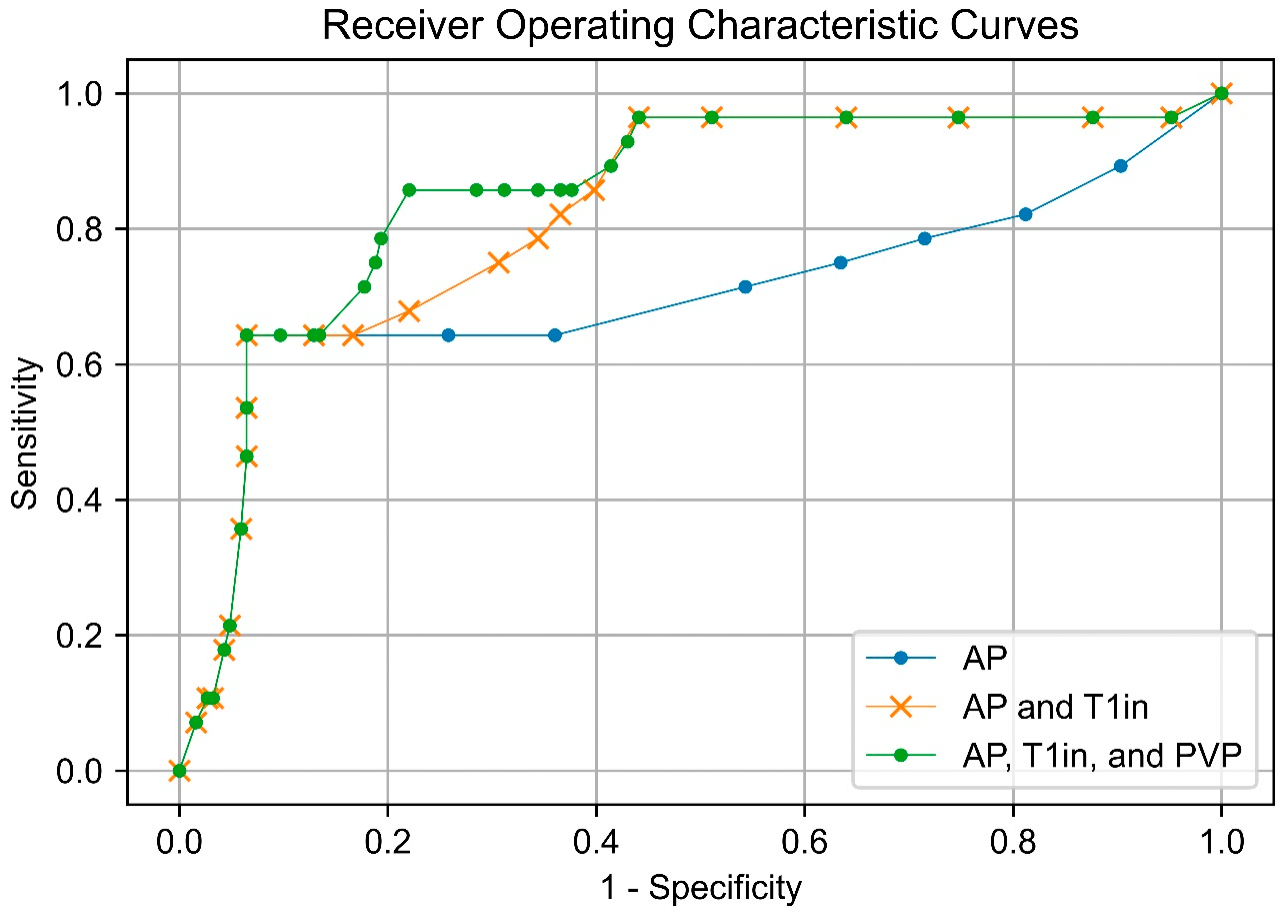
Receiver operating characteristic curve of the classification trees The AUC value of the combination of AP and T1in was 0.83 (95% CI 0.74–0.91) and significantly higher than that of AP (0.70, 95% CI 0.56–0.84, *p* = 0.003). The combination of AP, T1in, and PVP resulted in the maximum AUC (0.85, 95% CI 0.77–0.93) among all combinations of useful MR image types chosen by Fisher's exact test. However, there was no statistically significant difference between this and the AUC value of the combination of AP and T1in (*p* = 0.173). 95% CI, 95% confidence interval; AP, arterial phase image; AUC, the area under the curve; PVP, portal venous phase image; T1in, in‐phase T1‐weighted image

In contrast, fsT2WI was not included in the tree, although it had almost the same sensitivity and specificity as AP. This might be because fsT2WI had a stronger correlation with AP than T1in (Spearman's rank correlation coefficient = 0.64 between fsT2WI and AP and − 0.24 between T1in and AP). As a result, fsT2WI might not have provided additional information to that provided by AP in this study. However, this study included only resected HCCs, and their number was small. Therefore, further study is necessary. In addition, combining AP with fsT2WI properly may facilitate better differentiation. For example, fsT2WI can be used instead of AP when appropriate AP images are not available because of factors such as motion artifacts and inappropriate scan timing.

Thus, there is a large variety of MR image types that provide overlapping and sometimes contradictory information. CLTA revealed that in clinical practice AP is the most useful type of MR image and T1in the second most useful, among the eight useful types of MR images. There have been several studies that revealed useful MR image types for the differentiation between eHCC and pHCC,^8^, ^9^, ^24^ but how to diagnose with them has not been revealed. As far as we know, the present study has been the first to focus on this topic.

This study has several limitations. First, only lesions pathologically proven through surgery were retrospectively analyzed. This resulted in selection bias and imbalanced data. Second, because this was a retrospective study, the MRI machines and protocols were mixed. In particular, differences in b‐values on DWI and delay times on HBP might be an issue. However, we evaluated signal intensities qualitatively, and these differences would not have had a significant impact, as the results were consistent with previous studies.^8^, ^9^ Third, there was an insufficient number of cases, and we could not optimize the hyperparameters and the features. We made the tree as complex as we could without losing reproducibility. Therefore, the tree obtained would be the most complex one with reproducibility through this data. However, if there had been more cases, we could have created more complex trees, which would likely be clinically beneficial. Finally, further study is necessary to measure the true generalization performance of the tree obtained with different data.

In conclusion, CLTA revealed that in clinical practice AP is the most useful MR image type and that T1in the second in the differentiation between eHCC and pHCC. This is clinically important because it becomes possible to predict prognosis prior to treatment. In addition, there remains room for discussion on eHCC treatment policies. We may treat eHCC and pHCC in different ways in the future.

## AUTHOR CONTRIBUTIONS

**Fumihito Ichinohe:** Formal analysis (lead); investigation (equal); writing – original draft (lead). **Daisuke Komatsu:** Investigation (equal). **Akira Yamada:** Investigation (equal). **Takanori Aonuma:** Data curation (equal). **Ayumi Sakai:** Data curation (equal). **Shimizu Marika:** Data curation (equal). **Masahiro Kurozumi:** Data curation (equal). **Akira Shimizu:** Data curation (equal). **Yuji Soejima:** Data curation (equal). **Takeshi Uehara:** Data curation (equal). **Yasunari Fujinaga:** Conceptualization (lead); supervision (lead); writing – review and editing (lead).

## FUNDING INFORMATION

No specific funding was disclosed.

## CONFLICT OF INTEREST

The authors declare that there is no conflict of interest.

## ETHICS STATEMENT

This study was approved by our institutional ethics committee (no.2591). Informed consent was waived because this study was a retrospective study.

## Supporting information


Data S1

## Data Availability

The data that support the findings of this study are available from the corresponding author upon reasonable request.
