# Supplementary material for: Classification tree analysis to evaluate the most useful magnetic resonance image type in the differentiation between early and progressed hepatocellular carcinoma
Source: Cancer Med. 2023 Jan 22;12(7):8018–26. doi: 10.1002/cam4.5589 (PMC10134385; doi:10.1002/cam4.5589)
Supplement: Supplementary file 1 — Data S1 [file CAM4-12-8018-s001.docx]

Supporting Information: Magnetic Resonance Imaging Scanning Parameters

**a** MAGNETOM Trio

|  | T1in/T1op | ufT2WI | fsT2WI | DWI | Pre/AP/PVP  (FLTM) | Pre/AP/PVP  (FTM) | HBP |
| --- | --- | --- | --- | --- | --- | --- | --- |
| Repetition time  (ms) | 127–174 | 600–1200 | 1910–5373 | 3005–5005 | 216–233 | 2.5–2.7 | 3.0–3.5 |
| Echo time  (ms) | T1in: 2.46  T1op: 1.23 | 87–92 | 70–78 | 64–72 | 1.56–1.68 | 1.15–1.21 | 1.23–1.42 |
| Flip angle  (degrees) | 70–80 | 120 | 120–140 | 90 | 70–75 | 12 | 14–15 |
| Matrix size | 512  ×  288–416 | 384–448  ×  288–372 | 320–512  ×  236–360 | 256  ×  152–184 | 256–320  ×  152–220 | 384  ×  384 | 384–640  ×  240–460 |
| Slice thickness  (mm) | 5 | 4–5 | 4–5 | 4–5 | 5 | 4 | 3 |
| Field of view  (mm) | 420  ×  236–342 | 340–399  ×  255–332 | 319–400  ×  239–299 | 340–440  ×  212–288 | 400–420  ×  225–275 | 280–320  ×  280–320 | 380–429  ×  262–322 |
| b-value  (s/mm^2^) |  |  |  | 800–1000 |  |  |  |

*T1in/T1op* in-phase T1-weighted image/opposed-phase T1-weighted image, *ufT2WI* ultrafast half-Fourier acquisition single-shot turbo spin-echo T2-weighted image, *fsT2WI* fat-suppressed turbo spin-echo T2-weighted image, *DWI* diffusion-weighted image, *pre/AP/PVP* precontrast/arterial phase/portal venous phase image, *FLTM* fluoroscopic triggering method, *FTM* fixed time method, *HBP* hepatobiliary phase image

b MAGNETOM Prisma

|  | T1in / T1op | ufT2WI | fsT2WI | DWI | Pre/AP/PVP  (FTM) | HBP |
| --- | --- | --- | --- | --- | --- | --- |
| Repetition time  (ms) | 140-150 | 600-1200 | 2900-8574 | 1500-1600 | 2.7-3.4 | 2.7-3.4 |
| Echo time  (ms) | T1in: 2.46  T1op: 1.25 | 88-111 | 75 | 63-70 | 1.16-1.24 | 1.16-1.17 |
| Flip angle  (degrees) | 70 | 120 | 126-130 | 90 | 12-13 | 12-13 |
| Matrix size | 320  ×  240 | 384  ×  252-336 | 384  ×  276-324 | 256  ×  200-208 | 288-384  ×  260-300 | 288-384  ×  288-300 |
| Slice thickness  (mm) | 5 | 5 | 5 | 5 | 2.5-3.5 | 2.5-3.5 |
| Field of view  (mm) | 380-400  ×  285-300 | 380-400  ×  249-350 | 340-400  ×  244-304 | 380-420  ×  297-341 | 360-380  ×  297-380 | 380  ×  297-380 |
| b-value  (s/mm^2^) |  |  |  | 1000 |  |  |

*T1in/T1op* in-phase T1-weighted image/opposed-phase T1-weighted image, *ufT2WI* ultrafast half-Fourier acquisition single-shot turbo spin-echo T2-weighted image, *fsT2WI* fat-suppressed turbo spin-echo T2-weighted image, *DWI* diffusion-weighted image, *pre/AP/PVP* precontrast/arterial phase/portal venous phase image, *FTM* fixed time method, *HBP* hepatobiliary phase image

**c** MAGNETOM Vida

|  | T1in / T1op | ufT2WI | fsT2WI | DWI | Pre/AP/PVP  (FTM) | HBP |
| --- | --- | --- | --- | --- | --- | --- |
| Repetition time  (ms) | 150-180 | 500-1200 | 3371-6622 | 1600-2100 | 2.7-3.4 | 3.4 |
| Echo time  (ms) | T1in: 2.46  T1op: 1.23 | 88-111 | 76 | 50 | 1.25-1.44 | 1.19 |
| Flip angle  (degrees) | 70 | 120 | 130 | 90 | 12 | 13 |
| Matrix size | 288-512  ×  216-384 | 384  ×  276-336 | 384  ×  288-324 | 256  ×  208 | 288  ×  288 | 384  ×  300 |
| Slice thickness  (mm) | 5 | 5 | 5 | 5 | 3-3.5 | 2.5 |
| Field of view  (mm) | 380  ×  285 | 380-400  ×  285-333 | 360  ×  270-304 | 380  ×  309 | 360  ×  360 | 380  ×  297 |
| b-value  (s/mm^2^) |  |  |  | 1000 |  |  |

*T1in/T1op* in-phase T1-weighted image/opposed-phase T1-weighted image, *ufT2WI* ultrafast half-Fourier acquisition single-shot turbo spin-echo T2-weighted image, *fsT2WI* fat-suppressed turbo spin-echo T2-weighted image, *DWI* diffusion-weighted image, *pre/AP/PVP* precontrast/arterial phase/portal venous phase image, *FTM* fixed time method, *HBP* hepatobiliary phase image

d MAGNETOM Avanto

|  | T1in / T1op | ufT2WI | fsT2WI | DWI | Pre/AP/PVP  (FLTM) | Pre/AP/PVP  (FTM) | HBP |
| --- | --- | --- | --- | --- | --- | --- | --- |
| Repetition time  (ms) | 130–207 | 1100-1200 | 3112–6151 | 3119–7070 | 200–225 | 2.5–2.8 | 4.0–4.1 |
| Echo time  (ms) | T1in: 4.40  T1op: 2.20 | 89–92 | 76–80 | 61–65 | 2.02 | 1.08–1.20 | 1.82–1.89 |
| Flip angle  (degrees) | 80 | 150 | 150 | 90 | 90 | 13–15 | 15–16.5 |
| Matrix size | 320  ×  260–300 | 320  ×  240 | 384  ×  306–366 | 128  ×  84–96 | 320  ×  210–220 | 160–320  ×  160–320 | 320–352  ×  220–242 |
| Slice thickness  (mm) | 6 | 5 | 6 | 5–6 | 6 | 4–4.5 | 3 |
| Field of view  (mm) | 360–420  ×  308–393 | 360–420  ×  270–315 | 360–420  ×  286–400 | 400–470  ×  262–308 | 400–440  ×  262–302 | 300–340  ×  300–340 | 399–440  ×  274–302 |
| b-value  (s/mm^2^) |  |  |  | 800–1000 |  |  |  |

*T1in/T1op* in-phase T1-weighted image/opposed-phase T1-weighted image, *ufT2WI* ultrafast half-Fourier acquisition single-shot turbo spin-echo T2-weighted image, *fsT2WI* fat-suppressed turbo spin-echo T2-weighted image, *DWI* diffusion-weighted image, *pre/AP* precontrast/arterial/portal venous phase image, *FLTM* fluoroscopic triggering method, *FTM* fixed time method, *HBP* hepatobiliary phase image

e MAGNETOM Avanto fit

|  | T1in / T1op | ufT2WI | fsT2WI | DWI | Pre/AP/PVP  (FTM) | HBP |
| --- | --- | --- | --- | --- | --- | --- |
| Repetition time  (ms) | 150-250 | 600-1200 | 3692-7165 | 1500 | 2.7 | 3.3 |
| Echo time  (ms) | T1in: 4.76  T1op: 2.39 | 89-107 | 72 | 55 | 1.30 | 1.17 |
| Flip angle  (degrees) | 70 | 120 | 130 | 90 | 12 | 13 |
| Matrix size | 288  ×  232-248 | 320-384  ×  280-312 | 384  ×  324 | 256  ×  208 | 288  ×  288 | 384  ×  336 |
| Slice thickness  (mm) | 5 | 5 | 4-5 | 5 | 3.5 | 2.5 |
| Field of view  (mm) | 380  ×  306-327 | 380  ×  308-333 | 360  ×  304 | 380  ×  309 | 360  ×  360 | 380  ×  333 |
| b-value  (s/mm^2^) |  |  |  | 1000 |  |  |

*T1in/T1op* in-phase T1-weighted image/opposed-phase T1-weighted image, *ufT2WI* ultrafast half-Fourier acquisition single-shot turbo spin-echo T2-weighted image, *fsT2WI* fat-suppressed turbo spin-echo T2-weighted image, *DWI* diffusion-weighted image, *pre/AP/PVP* precontrast/arterial phase/portal venous phase image, *FTM* fixed time method, *HBP* hepatobiliary phase image

f Optima MR450w

|  | T1in/T1op | ufT2WI | fsT2WI | DWI | Pre/AP/PVP (FTM) | HBP |
| --- | --- | --- | --- | --- | --- | --- |
| Repetition time  (ms) | 184 | 4364 | 10000 | 15000 | 6.1 | 6.1 |
| Echo time  (ms) | T1in: 4.34  T1op: 2.11 | 87.80 | 88.45 | 65.90 | 1.67 | 1.67 |
| Flip angle  (degrees) | 90 | 90 | 160 | 90 | 12 | 12 |
| Matrix size | 512  ×  512 | 512  ×  512 | 512  ×  512 | 256  ×  256 | 512  ×  512 | 512  ×  512 |
| Slice thickness  (mm) | 6 | 6 | 6 | 6 | 4 | 4 |
| Field of view  (mm) | 380  ×  380 | 380  ×  380 | 380  ×  380 | 440  ×  440 | 440  ×  440 | 440  ×  440 |
| b-value  (s/mm^2^) |  |  |  | 1000 |  |  |

*T1in/T1op* in-phase T1-weighted image/opposed-phase T1-weighted image, *ufT2WI* single-shot fast spin-echo T2-weighted image, *fsT2WI* fat-suppressed fast spin-echo T2-weighted image, *DWI* diffusion-weighted image, *pre/AP* precontrast/arterial/portal venous phase image, *FTM* fixed time method, *HBP* hepatobiliary phase image
